# A recurrent mutation in *CRYBA1* is associated with an autosomal dominant congenital nuclear cataract disease in a Chinese family

**Published:** 2011-06-09

**Authors:** Guoxing Yang, Xinling Zhai, Jialiang Zhao

**Affiliations:** 1 Department of Opthalmology, Peking Union Medical College Hospital, Chinese Academy of Medical Sciences & Peking Union Medical College, Beijing, China; 2 Department of Opthalmology, 411 Hospital of the People's Liberation Army, Shanghai, China

## Abstract

**Purpose:**

Congenital cataracts are a clinically and genetically heterogeneous lens disorder. The purpose of this study was to identify the genetic mutation and the molecular phenotype responsible for the presence of an autosomal dominant congenital nuclear cataract disease in a Chinese family.

**Methods:**

Patients were given a physical examination and their blood samples were collected for DNA extraction. Genotyping was performed by microsatellite markers, and a logarithm of odds (LOD) score was calculated using the LINKAGE programs. Mutation detection was performed by direct sequencing.

**Results:**

Linkage to the crystallin beta A1 (*CRYBA1*) locus was identified. DNA sequencing of the gene revealed a c.279–281delGAG mutation in exon 4, which resulted in a glycine residue deletion at position 91 (ΔG91). This mutation was identified in all of the affected individuals but was not found in the 100 control chromosomes.

**Conclusions:**

Our results identify that the c.279–281delGAG mutation in *CRYBA1* is responsible for the autosomal dominant congenital nuclear cataract disease in this Chinese family.

## Introduction

Congenital cataracts are common and are the cause of approximately one third of infant blindness. They are found in approximately 1–6/10,000 live births. One quarter of congenital cataract diseases are hereditary [1-4]. Congenital cataracts are a clinically and genetically heterogeneous lens disorder. Cataracts that are phenotypically identical can result from mutations at different genetic loci and can have different inheritance patterns. Conversely, cataracts with dissimilar phenotypes may result from mutations in a single gene or gene family. It is believed that the type of genetic mutation is related to the morphology of the cataract [5]. To date, approximately 40 genetic loci have been linked to congenital cataracts, and 26 genes have been cloned and sequenced, including crystallins, connexins, heat shock transcription factor-4, aquaporin-0, and the beaded filament structural protein-2 [5].

Recently, a Chinese family in Northeast China has been identified with five generations of congenital nuclear cataracts. This pedigree is further characterized and identified in relation to the recurrent mutation c.279–281delGAG of crystallin, beta A1 (*CRYBA1*), which is associated with this eye phenotype.

## Methods

### Patients and clinical data

The five-generation family that enrolled in this study was located in Northeast China. A clinical examination, peripheral blood collection, and DNA extraction were performed on the participating family members, in the Department of Ophthalmology, at the Peking Union Medical College Hospital. The study was performed in accordance with the Declaration of Helsinki and approved by the Institutional Review Board and Ethics Committee of Peking City, and informed consent was obtained from all participants. The recruited family members included fourteen patients that were confirmed with congenital nuclear cataracts. Blood was collected from eight patients and one unaffected family member. The clinical data of these nine subjects was ascertained by detailed ocular examinations.

### Genotyping and linkage analysis

DNA was isolated from the blood samples of the nine subjects using the method of phenol-chloroform to extract the DNA. Fluorescently labeled microsatellite markers (Sangon Biotech Co., Ltd., Shanghai, China) were used for linkage analysis of 26 candidate gene regions. A two-point linkage analysis was performed with MLINK, from the LINKAGE program package.

### Mutation analysis

The coding exons of C*RYBA1* were amplified by a polymerase chain reaction (PCR) and a set of six pairs of primers (Table 1) [6]. Briefly, PCR amplification conditions were: Reaction Mixture Set Up (50 μl); 2 ml (40 ng/ml) of DNA, 1.5 ml (10 μM)) of each exon primer, 20 ml of water , and 25 ml of PCR mix (2× EasyTaq PCR SuperMix; TransGen, Beijing, China) in a final reaction volume of 50 ml. Thermal cycling conditions were: an initial denaturation step at 95 °C for 5 min, 38 cycles (30 min at 95 °C, 30 min at 52 °C, and 30 min at 72 °C) and a final 10 min 72 °C extension.The PCR products were sequenced on an ABI3730 automated sequencer (PE Biosystems, Foster City, CA).

**Table 1:** Primers used for *CRYBA1* amplification.

**Exon**	**Primer (5′-3′)**	**Product length (bp)**	**Annealing temperature (°C)**
CRYBA1-1F	GGCAGAGGGAGAGCAGAGTG		
CRYBA1-1R	CACTAGGCAGGAGAACTGGG	207	52
CRYBA1-2F	AGTGAGCAGCAGAGCCAGAA		
CRYBA1-2R	GGTCAGTCACTGCCTTATGG	293	52
CRYBA1-3F	AAGCACAGAGTCAGACTGAAGT		
CRYBA1-3R	CCCCTGTCTGAAGGGACCTG	269	52
CRYBA1-4F	GTACAGCTCTACTGGGATTG		
CRYBA1-4R	ACTGATGATAAATAGCATGAACT	357	52
CRYBA1-5F	GAATGATAGCCATAGCACTAG		
CRYBA1-5R	TACCGATACGTATGAAATCTGA	290	52
CRYBA1-6F	CATCTCATACCATTGTGTTGAG		
CRYBA1-6R	GCAAGGTCTCATGCTTGAGG	295	52

Cited from [6]

## Results

### Clinical findings

**Figure 1:**
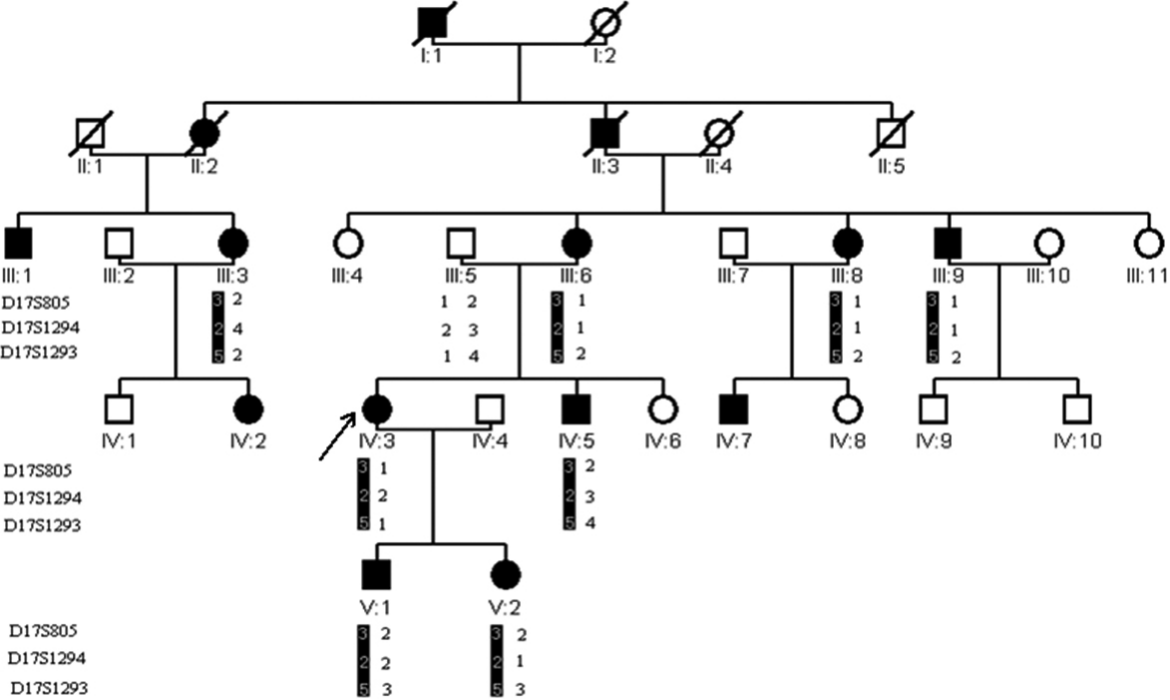
The pedigree and haplotype of the family. A five-generation pedigree with nine subject family members is shown. Three markers: D17S805, D17S1294, and D17S1293 that are adjacent to *CRYBA1* were selected to be analyzed. There was a cosegregation of the diseased haplotype (represented by the black bar) in eight patients of the family. The arrow indicates the proband.

**Figure 2:**
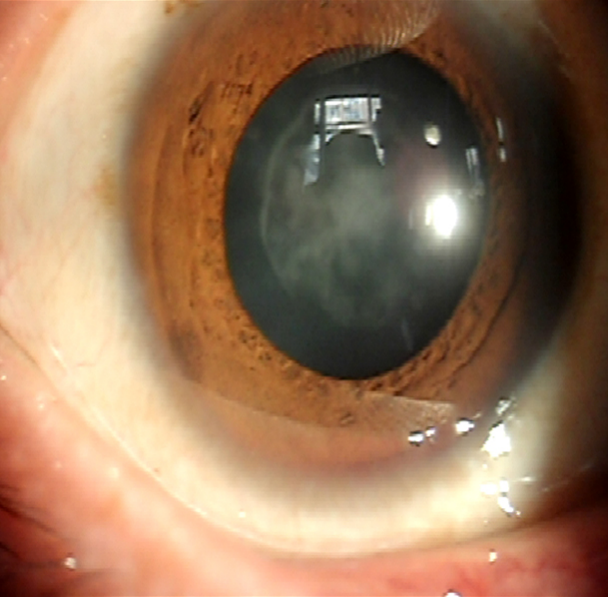
A slit lamp photograph showing the nuclear cataract of a patient III:8 (from Figure 1).

We had verified a five-generation family with fourteen confirmed individuals that had congenital cataracts (Figure 1 and Figure 2). Blood samples were collected from eight of the patients. All of the patients that participated have had no other clinically related ophthalmic syndromes.

### Linkage and haplotype analysis

Positive logarithm of odds (LOD) scores at 17q were obtained (Table 2). A maximum positive LOD score of 1.50 at θ=0.00 was obtained by marker D17S1293. There was a cosegregation of the haplotype in all eight of the affected subjects that were analyzed (in Figure 1).

**Table 2:** Result of linkage analysis.

** **	**LOD scores at θ=**					
**Marker**	**0.00**	**0.01**	**0.10**	**0.20**	**0.30**	**0.40**
D17S805	1.38	1.35	1.08	0.78	0.50	0.23
D17S1294	0.94	0.91	0.69	0.45	0.25	0.09
D17S1293	1.50	1.46	0.69	0.80	0.48	0.21

### Mutation analysis

**Figure 3:**
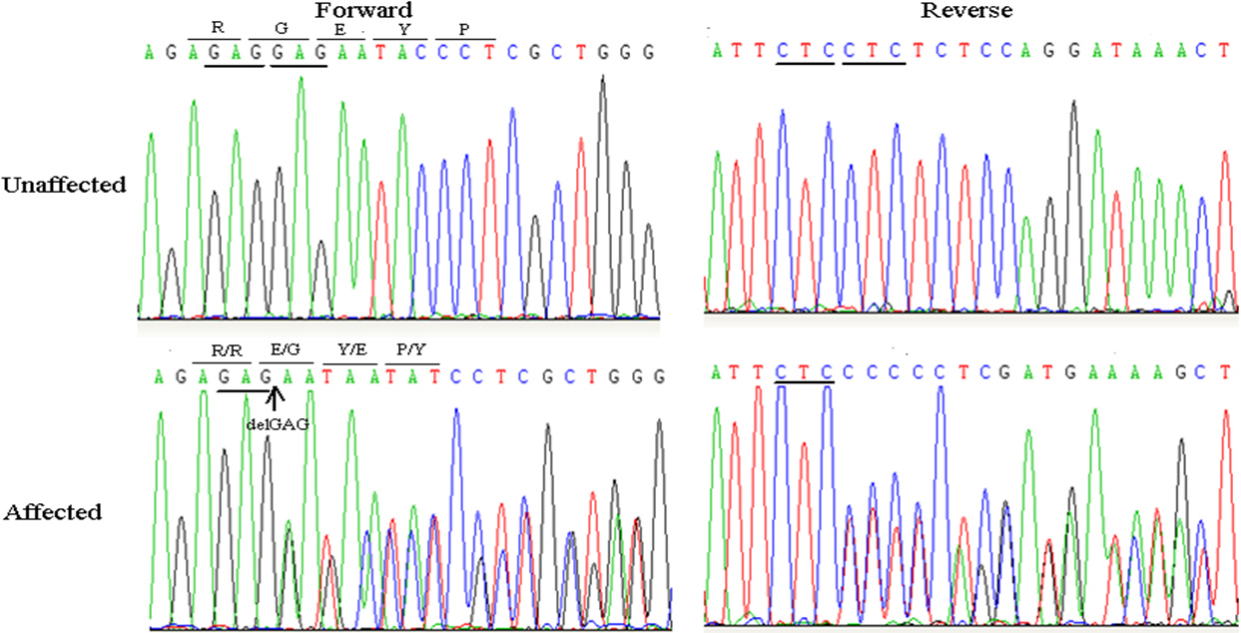
DNA sequences of *CRYBA1* in unaffected and affected individuals.

By direct sequencing of the *CRYBA1*coding region, a c.279–281delGAG mutation was detected (Figure 3). The mutation results in a glycine residue deletion at position 91(ΔG91). The cosegregation of the mutation was only found in the subjects and not in other members of the family, or in any of the 100 control chromosomes that were analyzed from the same ethnic background.

## Discussion

This study identified a recurrent mutation (c.279–281delGAG) in the *CRYBA1* gene of a five-generation Chinese pedigree with autosomal dominant nuclear cataract disease. *CRYBA1*comprises 6 exons that are alternatively spliced to produce 2 proteins (βA1- and βA3-crystallins). The βA1- and βA3-crystallins consist of seven protein domains: four homologous Greek key motifs, a connecting peptide, and NH_2_- and COOH-terminal extensions. These two proteins are identical except for 17 additional amino acid residues found on the NH_2_-terminal arm of βA3-crystallin.

In previous reports, three mutations have been identified in *CRYBA1* in multiple family and ethnic backgrounds, and have resulted in eye disease [6-15]. One previously identified mutation (c.279–281delGAG) is a 3 bp (GAG) deletion that occurs at the nucleotide positions 279–281, and results in an in-frame deletion of a glycine residue at position 91 (ΔG91). This mutation has been identified in an English family with lamellar cataracts [8], a Chinese family with nuclear cataracts [7], a Swiss family with suture-sparing nuclear cataracts [6], and two Chinese families with pulverulent nuclear cataracts and pulverulent lamellar cataracts [9]. Reddy et al. [8] studied the mechanism of the mutation of p.G91del in CRYBA1, and found that the mutant protein created defective folding and a reduction in solubility.

Two different mutations were identified that altered the same position in the third IVS (IVS3+1G>A and IVS3+1G>C), which caused the G residue to alter into either an A or C. The IVS3+1G>A mutation was detected in an Indian family with zonular sutural cataracts [10], an Australian family with nucleus and Y-sutural opacities [12], a Chinese family with posterior polar cataracts [14], a Chinese family with progressive cataracts [15],and two Indian families with zonular lamellar opacification [13]. The IVS3+1G>C mutation was present in a Brazilian family with pulverulent cataracts [11].

Since the mutations c.279–281delGAG and IVS3+1 have been found in multiple families, it suggests that these two locations are hot-point mutation sites.

In conclusion, the c.279–281delGAG mutation in *CRYBA1* is responsible for the pedigree of this Chinese family. It can be further argued that *CRYBA1*is responsible for autosomal dominant congenital nuclear cataract disease.
